# User Localization During Human-Robot Interaction

**DOI:** 10.3390/s120709913

**Published:** 2012-07-23

**Authors:** F. Alonso-Martín, Javi F. Gorostiza, María Malfaz, Miguel A. Salichs

**Affiliations:** Robotics Lab, Universidad Carlos III de Madrid, Av. de la Universidad 30, 28911 Leganes, Madrid, Spain; E-Mails: jgorosti@ing.uc3m.es (J.F.G.); mmalfaz@ing.uc3m.es (M.M.); salichs@ing.uc3m.es (M.A.S.)

**Keywords:** sound source localization, robot audition, social robot, array-microphone, phonotaxis, proxemics, dialog system

## Abstract

This paper presents a user localization system based on the fusion of visual information and sound source localization, implemented on a social robot called Maggie. One of the main requisites to obtain a natural interaction between human-human and human-robot is an adequate spatial situation between the interlocutors, that is, to be orientated and situated at the right distance during the conversation in order to have a satisfactory communicative process. Our social robot uses a complete multimodal dialog system which manages the user-robot interaction during the communicative process. One of its main components is the presented user localization system. To determine the most suitable allocation of the robot in relation to the user, a proxemic study of the human-robot interaction is required, which is described in this paper. The study has been made with two groups of users: children, aged between 8 and 17, and adults. Finally, at the end of the paper, experimental results with the proposed multimodal dialog system are presented.

## 1. Introduction

Animal survival mostly depends on their effectiveness in perceiving their environment. Using this sensorial information, they must be able to behave in order to maintain their needs satisfied by, for example, moving towards food or avoiding possible risks. Mobile autonomous robots, especially those designed to move in hostile environments, need to be equipped with similar abilities. On the other hand, for social robots, the perception of their environment is also used to improve their natural interaction with human beings during a conversation.

In nature, animals use their auditory sense for detecting the sound source; nevertheless, the number of “sensors” (ears) used in this process is not uniform. For example, there are some invertebrates, such as the mantis religiosa, with just one ear, and variable audible frequency [1].

In recent years, the research about how the human [2,3] and the animal [4,5] auditory systems work has been applied to robotics. Therefore, a new research area has emerged: “Robot Audition” [6–10]. The majority of the cited works focus on mobile robots with no social applications, so their main objective is to follow a sound source (phonotaxis) and not to situate at the right position during a dialog with a user (proxemics).

Social robots are designed to live among society; therefore, it is important that they can comply with communication rules such as the respect for personal spaces, among others. Moreover, it is also desirable that they have an advanced auditory system with features similar to those of the human being. Human beings have two ears to sense the sounds from the environment, and the brain, thanks to the phase and amplitude differences of the auditory signals, carries out the sound source localization.

The main goal of Robot Audition, applied to social robotics, is to improve the human robot interaction (HRI) during the dialog process. In this work, this dialog is possible thanks to the use of a complex system formed by several independent modules, working in a coordinated way, to obtain the most natural HRI. One of these modules, in our multimodal dialog system, is the sound source localization, which helps to situate the robot properly during the interaction (proxemics). This appropriate robot position with respect to the interlocutor depends on several conditions that will be analyzed later during the proxemic studies.

Maggie, our social robot, not only uses the auditory information to determine the sound source localization, but also others such as visual information and distance to user. In this paper we present a complex and modern multimodal dialog system, in which the sound source localization plays a very important role in the robot spatial situation.

This paper is organized as follows. Section 2 presents a review of the works related to the sound source localization problem, and to the study of the proxemics during the interaction between humans and between humans and robots. In Section 3, the social robot Maggie, which is our experimental platform, is introduced. Later, in Section 4, a proxemic study is performed to analyze the adopted allocation between user/s and Maggie during a natural interaction, which depends on several aspects (age, experience, gender, *etc.*). This collected information, together with that given by the User Localization module described in Section 5, is very useful to determine the most appropriate robot allocation during HRI. The experiments carried out in relation to the user localization are shown in Section 6. Finally, the main conclusions and future works are outlined in Section 7.

## 2. Related Work

### 2.1. Sound Source Localization Problem

An artificial auditory system may be used for three purposes: (1) sound source localization; (2) to separate the sound sources into different channels; (3) to extract sound features to perform different tasks such as speech recognition, emotion detection, or user identification. In this work we focus on the first point; the third one has already been analyzed in previous works [11].

In order to localize sound sources, according to the works presented in [12,13], there are two main approaches to this problem. One of them is to study the amplitude differences generated by a sound source among the microphones (or ears) used to perceive the signal. This method is the one used in this work, and it is based on the comparison of the volume differences between the microphones in order to determine the angular difference in relation to the sound source. The microphone closest to the sound source should receive the signal with the biggest amplitude in comparison to those received by the rest of microphones. It must be said that the accuracy of this method is greatly influenced by the reflection of the sound signal due to the objects present in the environment, such as walls and furniture. The second method consists of the analysis of the phase differences produced between the different signals received by each of the microphones related to the same sound source. This method is based on the idea that the same signal generated by the sound source will be perceived by the closest microphone before by the rest of them. The accuracy of this second method depends on the size and the relative position of the microphones: if the microphones are very close to each other, all of them will receive almost the same signal [14,15].

There is a third method, not so extended due to its complexity, that only needs one microphone to localize the sound source [16]. This method analyzes the differences in the spectrum produced by the same sound source from different positions in relation to the microphone. Human beings, with just two ears, are able to differentiate if a sound comes from the front or from the back. Moreover, we are able to determine if the sound is produced at the same distance and orientation. This is possible thanks to this third method which makes it possible for a person, with just one ear, to localize where the sound comes from. Nevertheless, for an artificial auditory system, based on one microphone, this is quite challenging since a previous knowledge of the sound is needed to be able to compare it with the sound received from another position. In human beings, this knowledge is acquired through life experience [17].

The combined use of the first and the second methods is becoming quite popular in robotics [6–8,10,18,19]. In order to do this, there are certain hardware specifications that limit its implementation: the microphones must be situated sufficiently far from each other to be able to capture phase differences between the received signals. This implies that the robot must have a minimum size to situate the microphones correctly. Commercial microphone arrays can also be found (http://www.acousticmagic.com/) with a minimum size of 11 inches, which can be inconvenient and not very aesthetic. Another additional problem is the necessity of having an acquisition board that simultaneously read all microphones.

In robotics, two basic software packages have been developed to cover the three purposes of the artificial auditory system previously described: ManyEars [20,21] and HARK [22,23]. In relation to the sound source localization, these frameworks implement particles filter algorithms [24] for localizing in a very robust way. These algorithms are based on the first and the second methods (amplitude and phase differences).

The main shortcoming of the use of these frameworks is their complexity; besides, since they use the phase difference method, we must also consider the hardware limitations: the microphones distribution and the need of a specific board for simultaneous microphone reading.

In our approach, in order to cover our necessities, and thanks to the implemented sensorial fusion, this complex solution is not needed. The approach followed is much simpler, based on amplitude differences and with no need for using an specific acquisition board.

### 2.2. Phonotaxis and Proxemics

First, let us define these two important concepts: phonotaxis and proxemics.

Phonotaxis can be defined as “the movement of an organism in relation to a sound source. For example, females are often attracted by the courtship song of a potential mate (*i.e.*, positive phonotaxis), or animals may flee from the sound of a predator (*i.e.*, negative phonotaxis).” (A dictionary of Biology: http://www.encyclopedia.com/doc/1O6-phonotaxis.html)

On the other hand, proxemics can be defined as the part of the semiotic (is the study of signs used in communication) devoted to the study of the spatial organization during linguistic communication. More precisely, proxemics studies the proximity, separation relations, *etc.*, between people as they interact [25]. Moreover, proxemics tries to study the meaning of these behaviors. This science allows people to create an interaction according to a spatial/temporal framework which expresses certain meanings and, in some occasions, with complex social restrictions related to sex, age, social background, culture, *etc.* Sometimes, the spatial distribution is established a priori, for example, in a courtroom or in a religious ceremony.

Some works on Robot Audition [6–10] just focus on phonotaxis. According to its definition, phonotaxis allows the user to determine where the attentional focus is, and then, to move towards it using only the auditory sense.

In our social robot, the main goal of the sound source localization system is not just to move towards or away from the user (phonotaxis), but to situate correctly during its interaction with one or several speakers simultaneously (proxemics).

### 2.3. Proxemic Studies during Human-Human Interaction

During the seventies a group of researchers, among them the anthropologist Edward T. Hall [25], applied the model that ethologists such as Huxley or Lorentz designed for the animal world to study the communication in human societies, and introduced the term Proxemics. Hall identified several kinds of spaces, among them, the one called personal space. This is the space created by the participants of a certain interaction, and varies as a function of the kind of meeting, the relationship between the speakers, their personalities, and other factors. Hall also proposed a model in which the personal space is classified in four subcategories:

1. Intimate space, from physical contact to 45 cm, approximately. This distance could be divided into two different intervals: between 0 and 15 cm, including physical contact; and between 15 and 45 cm, which corresponds to a less intimate distance, but still requires privacy.
2. Casual-personal space, from 45 to 120 cm. This is the common distance during interpersonal relations, and it allows the physical contact with another person.
3. Social space, from 120 to 346 cm. This is for situations where no personal questions are treated.
4. Public space, from 346 cm to about 7.6 m or more. At this distance the participants must amplify their voice to make the communication possible.

Hall notes that the model is based on his observations of a particular sample of adults and therefore, it cannot be generalized to all societies. It is obvious that there are different rules for every culture about the place and distance that must be maintained in certain situations, and also, this gives information about the social relationship between the participants. There is an adequate distance in every situation according to some rules established by the community. The participants know, or must learn, those rules in order to have successfully interpersonal relationships and to avoid possible conflicts or misunderstandings.

Other important works of that time [26,27] analyze and experiment with different factors that influence proxemics, such as the look, the personality, the number of people interacting, *etc.* Those works explain the necessity of eye contact during the interaction. Eye contact during more than 10 s increases anx0iety; however, the absence of a direct eye contact makes people feel out of the conversation.

In a very specific work, Lambert [28] determined communicative distance measures according to the kind of affective situation presented (see Table 1). However, he did not consider many other aspects also related to human interaction, which directly influence the spatial situation. Those factors will be analyzed in Section 4.

Human beings, as the rest of animals, manage the space around us and use distances as a way of satisfying fundamental needs. Moreover, proxemic studies have been able to establish that the perception we have about personal and social space is determined by our culture [29]. Ethologists claim that the human beings are territorial animals, that is, we select our favorite places and we get annoyed when somebody else comes to occupy them [30].

### 2.4. Proxemic Studies during Human-Machine and Human-Robot Interactions

Some studies have been done about human-machine interaction [31]. In those works, the users interact with computers or virtual agents (no embodiment); therefore, they cannot be extrapolated to the human-robot interaction research, since this interaction does not focus on the screen and mouse use.

The use of sound source localization in social robotics, and its utility in proxemics, is a much richer and ambitious task than just the phonotaxis skills implemented in general robotics. A correct implementation of a proxemic dialog allows the interlocutors to have a conversation at the right distance and orientation. These two measures vary depending on several features such as interlocutor identity, number of interlocutors, distance and orientation before the interaction, and affective state of the robot and the interlocutors. Moreover, even the appearance of the robot (height, weight, color, voice, tone) may influence proxemics [32]. All those user's characteristics can only be obtained during real interactions, if the robot has a complete multimodal dialog system with the sufficient modules capable of executing those extraction tasks.

In literature, we can find several studies about proxemics and HRI which analyze some of those factors. For example, Breazeal [33] found that humans socially responded to zoomorphic robots in non-verbal ways, and respecting the robot's interpersonal space. Besides, Httenrauch [34] observed that in HRI user trials most participants kept interpersonal distances from the robot corresponding to the second Hall's Personal Spatial Zone (0.45 to 1.2 m). Another research [35] found that participants generally allowed robots to approach more closely during physical interactions than under verbal or no interaction conditions.

Another approach related to the appearance of the robot was presented by Kheng in [36]. He related proxemics and user experience (short-time and long-time interactions) gained by living with the robot over a period of five weeks. During their first encounter, the participants exhibited a strong tendency to allow the robot with a mechanoid appearance to approach closer than the robot with humanoid appearance. Therefore, this work demonstrated that the approach distances for both kinds of appearances, humanoid and mechanoid, are significantly different. People generally preferred to be closer to mechanoid robots than to humanoid ones, although this tendency faded away as the participants got used to the robots.

In [32] severals factors were also analyzed. They found out that a robot looking at people's faces also influences proxemic behaviors. Besides, they found that women maintain larger personal spaces from robots looking at their faces than men. They learned how attitudes, personal experiences, and personality factors influence the proxemic behavior. People who are more agreeable (personality trait) move closer toward robots, unlike people who hold negative or more neurotic attitudes toward robots, who stand further away from approaching them. Following this same line, in [37] it is shown that participants who disliked the robot compensated the increase in the robot's gaze by maintaining a greater physical distance from it. On the contrary, participants who liked the robot did not differ in their distancing from the robot across gaze conditions.

While some non-verbal behaviors can be considered as optional elements to be added to a multimodal dialog system, there are very few works that really include proxemic skills in a complete dialog system [38]. Previous cited proxemic studies have focused on their use out from the dialog system, but in natural HRI, they are essential for a complete dialog system that takes into account user information and profiles, and could relate them to get a right proxemic disposition.

## 3. Our Social Robot, Maggie

**Figure 1.**
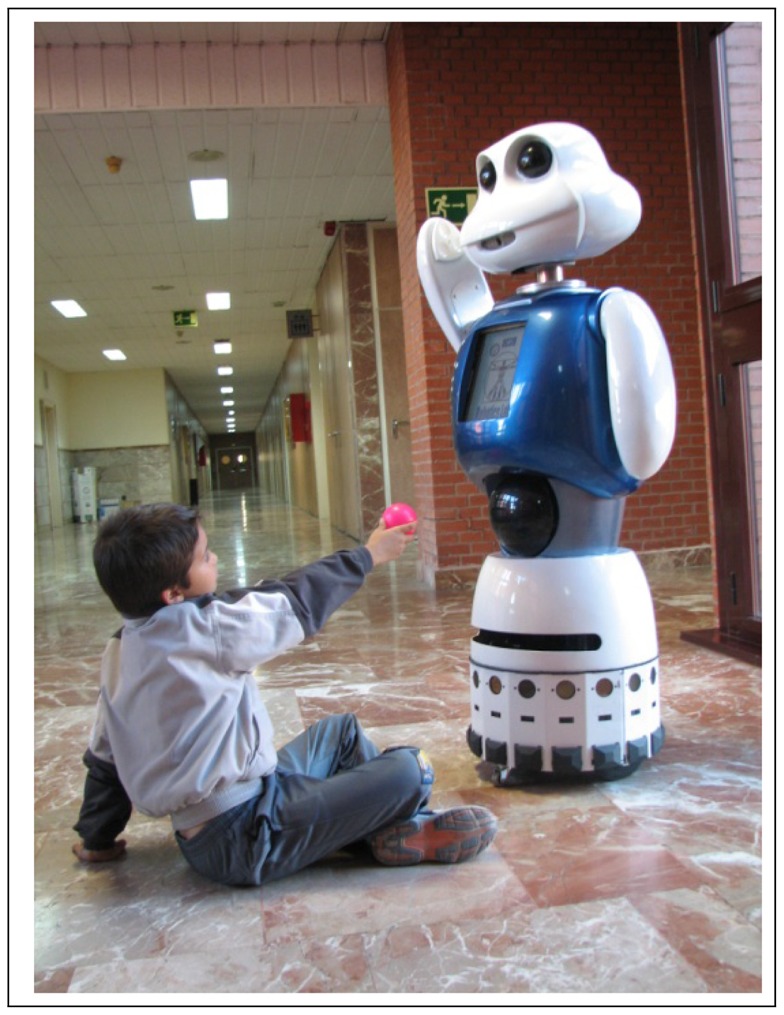
Our social robot, Maggie.

Maggie, see Figure 1, is a research platform for HRI studies [39]. The research focuses on finding new ways of improving robots in order to provide the user with new ways of working, learning, and having fun with them. Next, Maggie's software and hardware will be briefly described.

### 3.1. Hardware

Maggie is a social robot of 1.35 m high and it was designed to have an attractive appearance. This robot has a base with three wheels and it is also provided with 12 bumpers capable of detecting physical contact with the objects present in the environment. On the top of the base, there is an infrared laser telemeter able to precisely detect the distance to the nearest objects. Inside Maggie's “belly” there is a programmable infrared transmitter/receptor which allows the robot to control several devices such as TVs, Hi-Fi systems, *etc.*

The upper part of the robot is provided with several touch capacity sensors distributed over the surface like a “sensitive skin”. Moreover, there is a tablet-PC on the chest, so a bidirectional communication is possible between the user and the robot. Maggie also has two arms with one degree of freedom each.

Finally, the head is on the top, and it has two degrees of freedom. Inside the head there is a radio frequency identification (RFID) reader which is able to read RFID tags. Maggie's mouth is formed by a set of LEDs which illuminate while Maggie is talking. Moreover, there is also an onboard webcam (Logitech QuickCam Pro 9000), which gives us real-time images. In relation to the eyes, they have two animated eyelids giving Maggie a friendly appearance.

Recently, Maggie's physical skills have been extended thanks to the inclusion of a Microsoft Kinect (http://www.xbox.com/kinect). This powerful sensor is able to give color images and deep maps of the environment simultaneously. Moreover, it can also be used as a microphone to capture the user's voice, to follow people, and/or to obtain the user's pose within its field of vision.

The audio capture mechanism is based on a wireless earphone microphone, although we are currently experimenting with the Kinect and another microphone array integrated in Maggie's body. The robot can talk through the speakers incorporated in its neck.

Maggie is controlled by an onboard portable computer. The control architecture, which is described next, runs in this computer. It must be said that it is not necessary that the whole control architecture runs in this same computer. The distributed nature of the architecture allows any of its components to be executed by another computer.

### 3.2. AD, the Control Software Architecture

The Automatic-Deliberative (AD) architecture has been developed by the RoboticsLab (http://roboticslab.uc3m.es/roboticslab/) research group. The AD architecture is formed by two levels: the Automatic level and the Deliberative level. The low-level control actions are carried out at the Automatic level. The control primitives, which provide the communication and control of the hardware (sensors and motors), are set in this same level. The Deliberative level is formed by modules related to reasoning and decision making capabilities.

The main component of the AD architecture is the “skill”. A skill is an entity with reasoning capabilities, information processing and capable of carrying out actions. Moreover, one skill is also able to communicate with other skills at the same time. For example, the *laserSkill* manages the information given by the distance sensor, and it is able to make this information available to the rest of the skills that could need it. A more detailed description of the AD architecture can be found in [39–41].

## 4. Proxemic Studies with Maggie

As already stated in Section 2, Hall [25] presented studies related to proxemic research among human beings, mainly adults. Considering this and the four defined personal spaces, we have tried to adapt his experiments to two kinds of users: children and adults. The children, aged from 8 to 17, came from several schools and visited us separately in groups of 15, interacting with Maggie in sessions of 15 minutes. A total of 60 children were considered for this work. They interacted with the robot in different situations: in groups, one by one, and with/without help from an expert. Besides, none of them had previous experience with the robot. In the case of the adults, they were 5 members of our team, aged from 25 to 30. In this case, all of them had worked with Maggie previously and were aware of its functionalities.

The objective of these studies is to identify how those Hall's personal spaces relate to different kinds of users and situations. This information is essential for the dialog system (specifically for the dialog manager) to make the decision of placing the robot in a suitable position in relation to the user/s. In other words, to adapt the interaction distance to the kind of user/s.

All these interactions were recorded to analyze different factors that could influence proxemics during the interaction between the users and Maggie. During all these interactions the users were able to freely interact using all the dialogs and interaction possibilities offered by Maggie, so there were no restrictions of use. In the case of the children, each interaction was absolutely different, and they usually tried to play with the robot (an extensive description of robot games can be found in [42]). As previously said, the aim of this study is to determine the most suitable interaction distance for a certain kind of user. Therefore, the analysis of other factors such as dialog turns, interaction time, number of right responses, *etc.*, is out of focus in this work.

### 4.1. Interaction with or without Profiles

In this work, we are going to consider that there are two types of interactions with a social robot: without and with user profile. In the first one, the user interacts with the robot without having a profile, typically because that is the user's first interaction (maybe during a show or a demo). Therefore, the robot behavior focuses on catching the user's attention. If the user wants to interact with Maggie several times, it is convenient that they enroll in the system, so that the robot can adapt its behavior to the user profile. In the profile several features are stored: age, name, gender, experience, and language.

As mentioned, during an interaction with a user profile, the dialog can be adapted to the user, and one feature that needs adaptation is the interaction distance. Before loading the user profile it is necessary to identify the user. In our dialog system, the user is identified by his voice. In order to do that, during the enroll dialog the system learns the specific features of the user's voice (voiceprints) and saves them in a file that will be used for user identification.

In [32], the relationship between the user experience with the system and the proxemics is discussed. In fact, we have observed that for a user with a profile, it is quite common to maintain an interaction distance which corresponds to Hall's personal space 3 (120 to 364 cm). However, during a spontaneous interaction, when the robot is doing a show or the user is not experienced (without a user profile), the interaction distance corresponds to space 4 (public space).

Finally, it has been proved that, in interactions between humans, the familiarity range seems to be related to a lower interaction distance [25]. However, considering the experience gained with the robot as a measure of familiarity, we have observed that the interaction distance grows as the experience of the user increases. This seems to be due to Maggie's nature.

In order to communicate with Maggie, the speech recognition is carried out through a wireless auricular earphone, and the sound source localization is done by the own microphones of the robot. Therefore, as the experience increases the users realize that Maggie is able to hear them from 3 m away or more, since the audio signal goes from the emitter (user) to the receiver (robot). However, a novel user tends to be closer to Maggie trying to be better heard by the robot. In fact, when the speech recognition system does not understand what the novel user is saying, then they try to be even closer to Maggie and speak louder.

These conclusions have been observed during sessions where the children and the team members interacted separately with the robot.

### 4.2. Age

**Figure 2.**
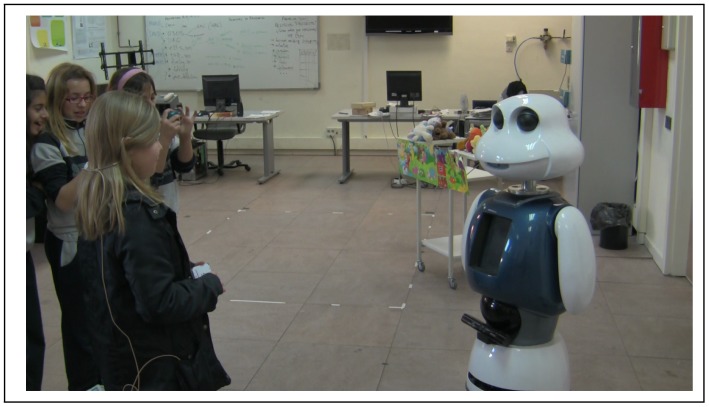
Interaction with children aged from 8 to 10.

**Figure 3.**
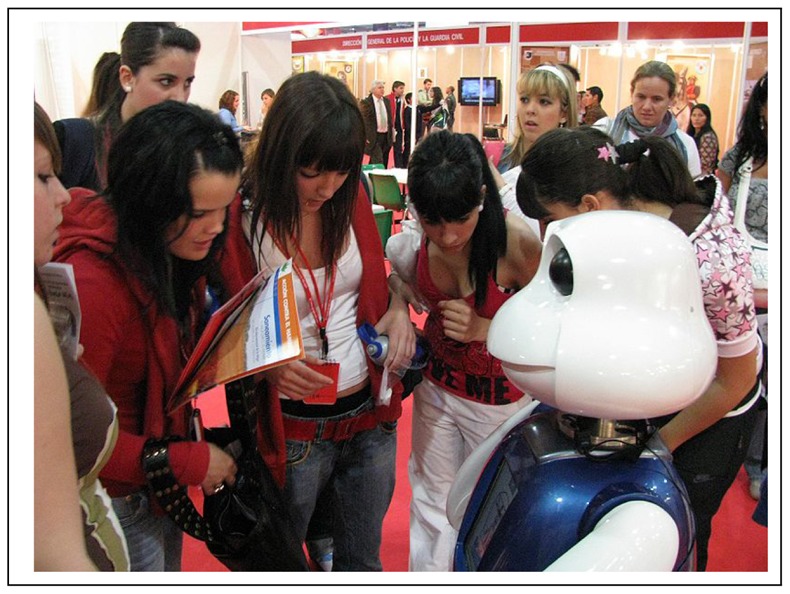
Interaction with children aged over 10.

Analyzing the collected data, we observed that age also influences the interaction distance. In average, in the interaction with children from 8 to 10 years old, the normal distance to the robot is bigger than 2 m (personal space 3), see Figure 2. However, children over 10, and adults, decrease the interaction distance to about 1 m, see Figure 3. We think that children under 10 years old feel more intimidated by the robot than older children. Children over 10 years old feel more curious and try to interact more closely with the robot. In the case of the adults, since they have already interacted with Maggie in other occasions, they feel more comfortable.

### 4.3. Personality

Personality is a factor that influences proxemics as shown in [43]. In our experiments, we have observed that, when the group of children is interacting with Maggie, those situated closer to the robot are the most extrovert ones. On the contrary, the shy ones tend to keep a certain distance to the robot, always looking at their teacher.

The most extrovert children interact with Maggie more enthusiastically, trying to catch its attention over the rest of children. It is obvious that it is difficult to measure the personality of the children, but in relation to HRI, it seems that the shyness degree is related to the interaction distance and its duration.

### 4.4. Gender

Another factor that could influence proxemics is the gender of the user. According to [44], it seems that women prefer to be in front of the robot and men at the side. However, in our studies, we could not corroborate this statement, since no significant differences between the boys' and girls' behaviors were obtained.

### 4.5. Number of Users

Although the dialog system is designed to interact with one user, that is, it is not possible to load more than one profile at the same time, any user can actually talk to the robot and perform some interactions in a cooperative mode. For this reason it is interesting to study the interactions in groups.

**Figure 4.**
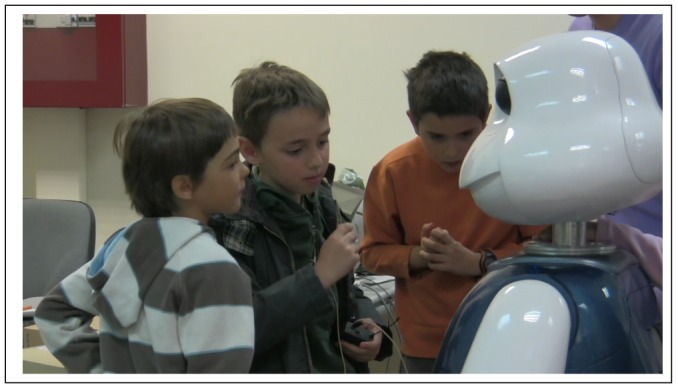
Groupal interaction.

We have observed that, during interactions with more than one child, the children tend to be very close to Maggie trying to catch the robot's attention separately. In fact, it has been observed that the same child who started to interact alone with the robot (situated far from it) approaches Maggie when more classmates are included in the interaction (see Figure 4).

**Figure 5.**
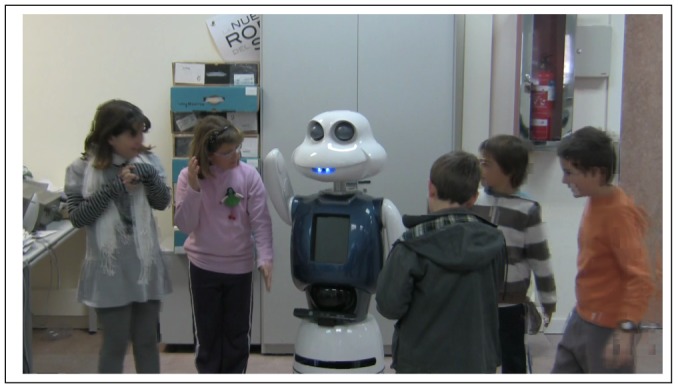
Children mimic robot dance.

Besides, coordination tasks have also been observed, for example, with the musical and dance robot skills. In those situations the children, with no external advice, tend to align themselves with Maggie and imitate its dance steps (see Figure 5) and therefore, their allocations with respect to the robot change.

### 4.6. Proxemic Rules for User-Maggie Interaction

In this section, the extracted set of rules in relation to the interaction distance between the users and the robot is presented. Those proxemic rules must be applied into our dialog system. Nevertheless, not all the analyzed factors can be applied to the current implementation. The user's personality could not be taken into account due to lack of tools to observe it. Moreover, the gender has not been considered since we did not find significant variations in the interaction distance with men and women. Finally, the number of users during the interaction has not been taken into account, since we are only able to load a user profile at a time.

**Figure 6.**
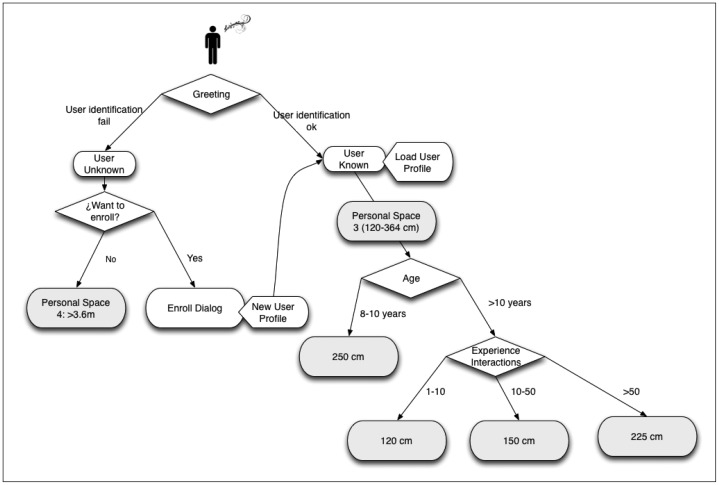
Proxemic rules.

In Figure 6, the proxemic rules applied during the HRI are shown. As can be observed, when the user greets the robot, two situations can emerge: that the user is identified (the robot has a user profile), or not (the robot does not have a user profile). In the first case, the robot loads the user profile and the user experience increases in one point. On the other hand, if the user is unknown, the robot asks them to enroll in the system. In the case that the user does not want to create a profile, the robot maintains its position at 3.6 m or more (personal space 4) from the user. On the contrary, if the user wants to register in the system, a user profile is created. In this case, or if the user already has a profile, the robot, as an initial position, situates between 120 to 364 cm (personal space 3). From this point and depending on the age of the user and the experience, this distance varies (always within the personal space 3). If the user is aged between 8 and 10, the distance is about 250 cm; otherwise, depending on their experience (measured by the number of interactions), the distances vary from 120 to 225 cm.

## 5. User Localization System

In order to implement the user localization ability in the multimodal dialog system of our robot, it is necessary to make a hardware and software description of the problem.

### 5.1. Hardware System: Used Sensors

An artificial sound source localization system, with just two microphones, is certainly imprecise. It is difficult to differentiate if the sound comes from the front or from the back, and also, to get a high level precision. However, a robot is not limited to use two microphones. In this sense, we have decided to use eight microphones placed around Maggie's base top in order to get a better approach to the localization ability of the human auditory system. Moreover, this allocation improves the robustness to noises.

**Figure 7.**
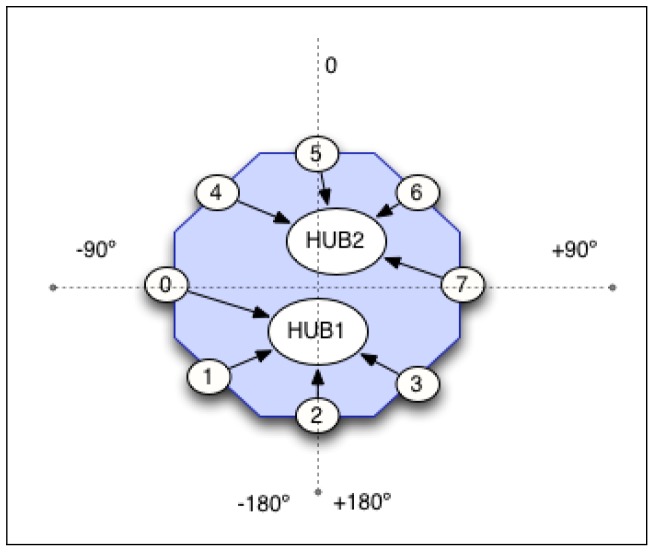
Microphone layout in the robot Maggie.

**Figure 8.**
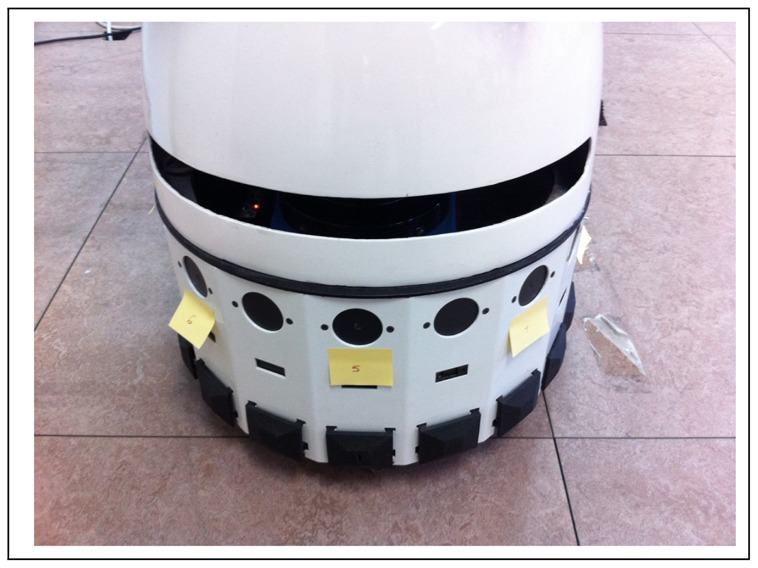
Microphones in the robot Maggie.

The eight directional microphones are connected to the computer through USB ports using two hubs. These microphones are placed on the base of the robot, at 21 cm high, forming a perfect circumference of 40 cm radius, see Figures 7 and 8.

In order to extract the sound features needed for the speech recognition, emotion detection, and user identification, we use an additional microphone, a directional auricular wireless microphone, much less exposed to the environmental noise [11].

The placement of the microphones in the lower part of the robot has been decided due to two reasons: first, because they are far from the speakers of the robot located in the neck; and second, because the circular shape of the base of the robot favors the calculations of the sound source localization algorithms.

It is important to notice that placing the microphones in the own structure of the robot favors the sound source localization task, since the body acts as a barrier for the audible waves which do not come directly to the closest microphone.

The farthest microphones from the sound source receive a lower intensity signal than those located in front of the source.

Moreover, the audio devices associated to the microphones must always be charged in the same order to avoid their logic disorder in the Operative System (OS) that manages their signals.

As already said, the user localization system not only uses the sound as the unique information input but also relies on visual information and distances to lower the error made by the auditory system. In order to do this, we use an infrared telemeter laser, which gives us information about distances, and the Kinect vision system, as shown in Section 3.

### 5.2. Software System

The AD architecture runs over a Linux OS, more precisely Ubuntu 11.10, and a sound architecture ALSA.

First, a calibration phase has been necessary. Each of the microphones has a different capture level (intrinsic gain), although all of them belongs to the same model. Therefore, it is necessary to fix (by trial and error) a uniform capture volume for all of them in the OS. Moreover, we must decide the sound intensity level that corresponds to a sound coming from a source situated close to the robot, and the average threshold of the human voice in order to differentiate it from the background noise.

The audio volume is sequentially checked over each microphone, at each iteration. For this reason, it is necessary to read a low amount of frames (256 is a good value), in order to maintain the blocking reading for each microphone as low as possible. In every iteration, 256 frames are read from each microphone. This iteration is so fast that the reading is quite similar to a simultaneous reading (less than 30 ms).

Over these frames, using ALSA functions (The Advanced Linux Sound Architecture (ALSA) provides audio and MIDI functionality to the Linux operating system), we calculate the sound intensity level reached by each microphone. This process is repeated during the reading of a certain number of iterations, five in our case, and we calculate an average value of the sound intensity read by each of the microphones. If we use a higher number of iterations, instead of five, the system would be less reactive to audible changes in the environment, since the average calculation will take more time than for a lower number of iterations.

Once we have calculated an average intensity value for every microphone during a set of fixed iterations, we check which microphone is the one that registers the highest intensity level. If this intensity level exceeds a certain threshold, previously fixed to filter voice or any other relevant sounds from the background, and the robot is not talking (since its own voice could be the sound source), then we determine that the orientation of the sound source is the same as the selected microphone.

The process described is specified in Algorithm 1.



**Algorithm 1** Sound source localization algorithm
**Require:**
*numMicrophones* =8, *numSamples* =256, *numIterations* =5, *voiceThreshold* =11001:int frames[numMicrophones][numSamples]2:int accumulatedVolume[numMicrophones]{The volume is computed or each microphone in several iterations}3:**for**
*numIter* ← 0 to *numIterations*
**do**4: readAudioSamplesAllMicrophones(frames)5: **for**
*numMicro* ← 0 to *numMicrophones*
**do**6:  **for**
*numSample* ← 0 to *numSamples*
**do**7:   accumulatedVolume[numMicro] += frame[numMicro][numMuestra]8:  **end for**9: **end for** {Look for the microphone with more accumulated volume}10: int microphoneWin = getMaximo(accumulatedVolume) {If robot is not speaking and accumulated volume of microphoneWin is upper the voiceThreshold}11: **if** (*accumulatedV olume*[*microphoneWin*] ≥ *voiceThreshold*) AND robotIsQuiet() **then**12:  int angleSoundSource = (360/numMicrophones)*microphoneWin13:  emit(angleSoundSource)14: **end if**15:**end for**


After the sound source localization system determines the orientation of the user, the localization system based on laser measurements starts to work. This system allows the robot to move forward/backward and is capable of measuring the interaction distances in relation to the user(s), providing much more precision. This allows the robot to move closer to or away from the user with high accuracy.

The laser, which is on-board Maggie, provides a cloud of points that corresponds with the distance between the objects around and the laser sensor. Using this information, the robot chases the cloud of points that matches the user's legs. The exact distance and orientation to be maintained between robot and user are provided by the dialog manager (the brain of the dialog system), based on the proxemics studies about Maggie (shown in Section 4), the information given by the user localization module (described in Section 6), and the user profiles.

Looking at the human behavior during a natural voice interaction, the process followed is quite similar. First, we use the auditory system to approximately localize the orientation of the sound source (the interlocutor), and to turn towards that orientation. Once the interlocutor is within our field of vision, the vision system is used to determine the distance and the precise orientation of the speaker. In our opinion, it is not necessary to have a very heavy and expensive user localization system based only on audible information, since our system is included inside a multimodal dialog system.

### 5.3. Implementation of the User Localization Skill in the Multimodal Dialog System

As previously said, the user localization system is included in a very complete and complex multimodal dialog system which controls the dialog flow, and so the HRI. This multimodal dialog system controls a huge number of features that must be considered during the dialog, such as the speech recognition, speech synthesis, gestures generation, emotion recognition, *etc.* Another characteristic also controlled by the multimodal dialog system is the one related to proxemics between the user and the robot.

**Figure 9.**
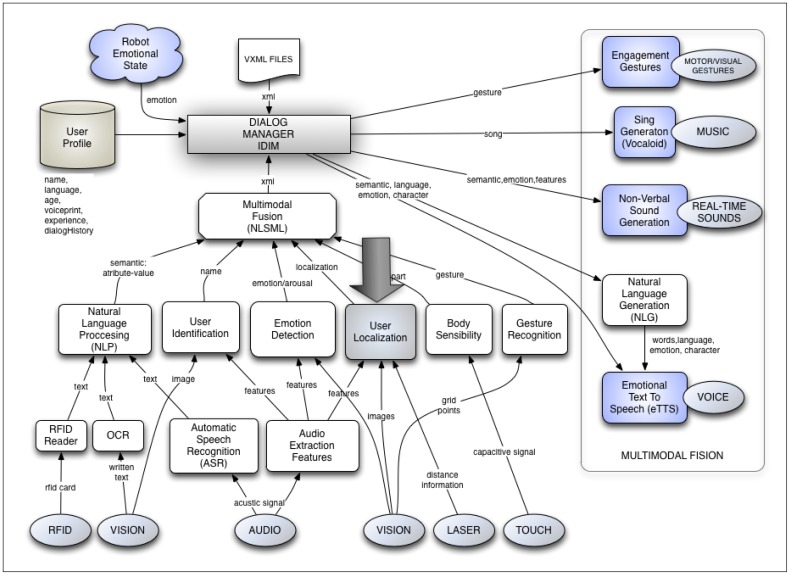
Multimodal dialog system in AD.

The sound source localization system described in the previous section is implemented in the AD architecture using the “User localization module”. In Figure 9 the complete multimodal dialog system is presented as well as this module. As can be observed, this module not only receives the auditory input, but also the visual and distance information. Then, it is able to do a multimodal fusion of all this information and get a greater precision in user localization than the one obtained using only the auditory information. Actually, for some authors, this cannot be considered as multimodal data fusion since, according to them, this is the synergistic combination of multi-thread flow of data from multiple heterogeneous sensors to provide more reliable and accurate information (see [45]). Instead, our module makes a sequential use of sensors.

The information processed and fused from the user localization module is given to the Multimodal Fusion Module. This module organizes all the information received by the rest of modules in a “macro-package” of processed information, which is formally sent to the dialog manager (IDiM) in an *xml* text file. Conceptually, this process corresponds to what is called “communicative acts” [46–48].

The dialog manager is the one that, using the processed sensorial information and adding the user profile (age, language, experience, name, and dialog history), can make intelligent decisions related to the spatial location of the robot in relation to the user.

Each of those modules is a skill in the AD architecture. Each of these skills can communicate with the rest of skills in two ways: by passing messages (events) or by using a shared memory (known as blackboard paradigm).

## 6. User Localization Experiments

### 6.1. The Sound Source Localization Module

In this section, we first present some experiments made only with the sound source localization module (using just the auditory input) to determine its reliability degree. If the precision of the sound localization is not good enough, it is quite difficult for the multimodal dialog system to determine a right spatial position during the interaction.

The room where the experiments were carried out is 11.40 × 6.20 × 3.20 m, with a medium reverberation due to the lack of furniture. In order to evaluate the sound source localization, a special group of people is not needed; therefore, the system has been tested by one member of our research team. In this case, the user was situated at different distances from the robot, between 0.5 and 3 m. We did not find significant variations of the accuracy of the results when the position of the user varies within the range of 0.5 to 3 m. This is because the capture volume of the microphones situated on-board Maggie is adjusted in such a way that they are able to correctly perceive a normal human voice tone at those distances. The obtained results are the following:

- Error average value in sound source localization: 23.72°
- Standard deviation in sound source localization: 25.82°

These values may seem to be high, but we must consider that the final User Localization module uses multimodal fusion, not just the auditory input. The major source of accuracy loss in a real environment is the appearance of undesired sounds, for example: the own sound generated by the robot when it moves or speaks, or even by its inner fans. In order to decrease the incidence of these problems we are currently working on echo cancellation techniques and noise active reduction.

### 6.2. The User Localization Module

**Figure 10.**
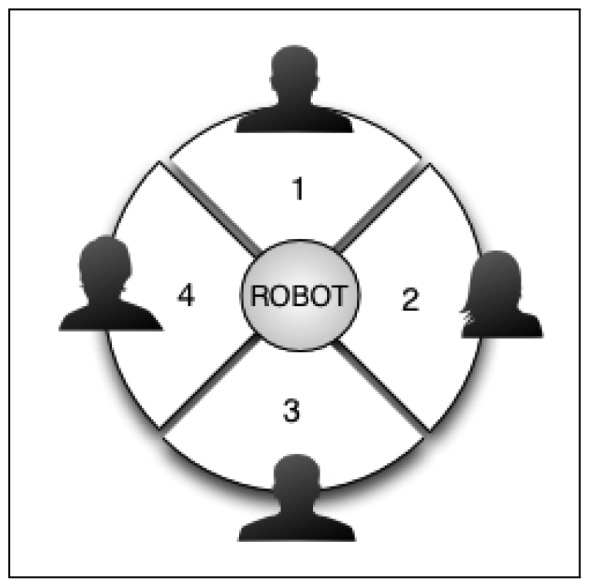
Localization Areas.

In order to prove the usefulness of the User Localization module in the multimodal dialog system, some experiments were carried out to check if the robot moved correctly toward or away from the user. For that purpose, the space around the robot was divided into four zones, see Figure 10. In each zone a different user is located at an initial distance between 0.5 and 3 m to the robot. The users were four team members already registered in the system with different profiles: ages between 25 and 30, three men and one woman, and experience values between 2 and 150.

**Figure 11.**
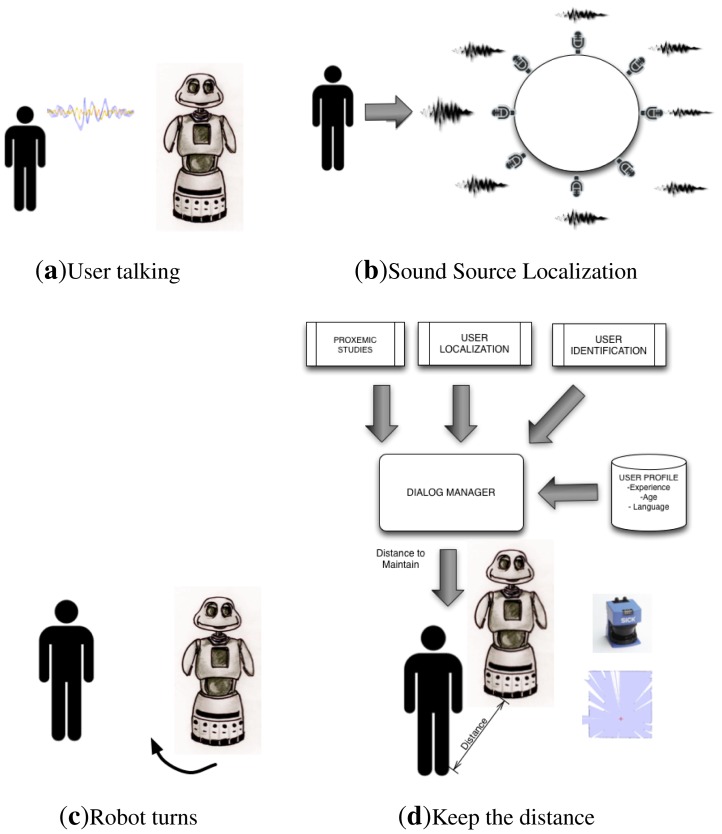
User Localization steps. (**a**) The user starts to talk to Maggie; (**b**) The User Localization System, using the received sound signals from the microphones, decides the angle of the user; (**c**) Maggie rotates toward the user position; (**d**) The Dialog Manager based on the observations from the proxemics studies (see Figure 6), the user localization information (with the laser sensor), and the user profile (load later to identify the user) gives commands to the motors of the base to maintain the proper distance to the user.

The dialog during the HRI starts by the user greeting the robot. Then, after detecting the approximate orientation of the sound source (the user), the robot must turn the right angle and move toward the user, maintaining a certain distance decided by the Dialog Manager. The precise interaction distance depends on the proxemic studies and the user profile, as has been detailed in Section 4.6. During the HRI, the dialog system checks periodically the user location and, if the interaction distance varies considerably (about 0.5 m with respect to the ideal computed distance), the robot moves to the proper allocation again. Note that the user was able to change position during the interaction, but the robot changes its position only if the interaction distance changes considerably. This process is repeated for each of the four users located in the four zones. An intuitive graphical description of the process can be seen in Figure 11.

The results obtained with the user localization system (the sound source localization and approximation using the telemeter laser) achieved a success of 87%. This means that, if there is one user in each zone, and one of them begins to speak, the 87% of the times Maggie turns and moves to the correct interaction zone (standard deviation of 12%) and maintains the proper interaction distance. Those results could be generalized to the other group of users, the children, according to the rules described in Section 4.6; however, it would be very interesting to verify them, evaluating the system with them in a near future.

It is important to note that the errors are mainly caused by two factors: failure in the sound source localization system (auditive systems) and/or errors in chasing the user's leg, since sometimes the cloud of points is lost or confused with another nearby object of similar shape. If the sound source error is not very high, it can be corrected by the laser system, since it is able to chase the user although he/she is not placed centered to the robot. Therefore, the main source of error is in the distinction between objects, which are close to the user and the robot, and humans. Currently, we are working to fix these problems, trying to distinguish between stationary objects (typically barriers) and moving objects (typically human).

## 7. Conclusions and Future Works

The user localization system in addition with the proxemics research we carried out with real users interacting with Maggie, have provided a proxemic ability to the multimodal dialog system presented here. This ability is the one responsible for positioning Maggie at the right place during the HRI phase, making this process much more natural. In this sense, the dialog system is able to adapt and to position the robot at the most appropriate distance for each communicative situation. Both tasks outlined in this paper are required to achieve this task: the user localization module, and the promexics study.

In order to localize the user, the robot first computes the position of the user using the sound source localization system, which makes use of eight microphones.

The goal interaction distance is determined according to the extracted rules obtained from the proxemic study, depending on the type of user, that is, their age, experience, *etc.* Once the robot has turned itself to face the user, it positions itself at the goal distance from the user. The laser is used to determine the approximate distance of the user and to maintain it close to the goal one.

In a near future, it is expected to get a greater sensorial fusion between the information provided by the stereo vision and the multimodal dialog system. Therefore, Maggie will add to its dialog system, based on voice and sounds, the benefits of the stereo vision.

In this paper, we have analyzed several factors related to the user which influence proxemics. However, other factors related to the robot remain to be studied and tested, such as the shape of the robot, colors, appearance, voice volume, weigh, *etc.* In this work we could not analyze them, since we only used one robot for the experiments. We are currently building new robots using the same dialog system, but with very different shapes. This will allow us to study how those external factors influence proxemics in a near future.

Moreover, it would be very interesting to include a more flexible customization of the interaction distance (personal space). For example, if a user feels uncomfortable at the interaction distance computed by the system, this distance would be marked as not suitable for this given user. It would be very desirable that the user could change her distance dynamically and naturally, using our dialog system.

## Figures and Tables

**Table 1.** Human-Human Personal Space Zones.

**Range**	**Situation**	**Personal Space Zone**
0 to 0.15 m	Lover or close friend touching	Intimate Zone
0.15 to 0.45 m	Lover or close friend only	Close Intimate Zone
0.45 to 1.2 m	Conversation between friends	Personal Zone
1.2 to 3.6 m	Conversation to non-friends	Social Zone
*>*3.6 m	Public speech making	Public Zone
